# Une petite malformation vasculaire provoquée

**DOI:** 10.11604/pamj.2015.21.72.3689

**Published:** 2015-05-29

**Authors:** Rajaa Elouarradi, Hatim Droussi

**Affiliations:** 1 Université QuadiAyyad, Service de Dermatologie à l'Hôpital Ibn Tofail, Centre Hospitalier Universitaire Mohammed VI, Marrakech, Maroc; 2 Université QuadiAyyad, Service de Chirurgie Plastique à l'Hôpital Ibn Tofail, Centre Hospitalier Universitaire Mohammed VI, Marrakech, Maroc

**Keywords:** Malformation vasculaire, tuméfaction non douloureuse, embryonnaire, vascular malformation, painless swelling, embryonic

## Image en medicine

Nous allons rapporter l'observation d'une patiente âgée de 22 ans, enceinte de 4 mois, avec notion de sensation d'un corps étranger au niveau de la pulpe du quatrième doigt de la main gauche manipulée par une aiguille, ce qui a donné naissance à cette tuméfaction non douloureuse (A, B). Cette lésion est non battante, non douloureuse, le reste de l'examen physique est sans anomalie. Il s'agit d'une rupture provoquée de malformation vasculaire à bas débit veineuse ou capillaro-veineuse, jamais diagnostiquée, et confirmée par l'echo-doppler. Les malformations vasculaires correspondent à une dysplasie d'origine embryonnaire développée au dépend du système vasculaire, elles sont congénitales, présentes à la naissance ou apparaissent quelques mois ou années après, et ne régressent jamais. Ce type de malformation est influencé par les phénomènes hormonaux, il existe souvent une aggravation au moment de la puberté et lors des grossesses. Une abstention thérapeutique a été proposée chez cette patiente avec surveillance rapprochée au cours de la gestation, vu le risque de saignement. Un traitement chirurgical peut être envisagé ultérieurement.

**Figure 1:**
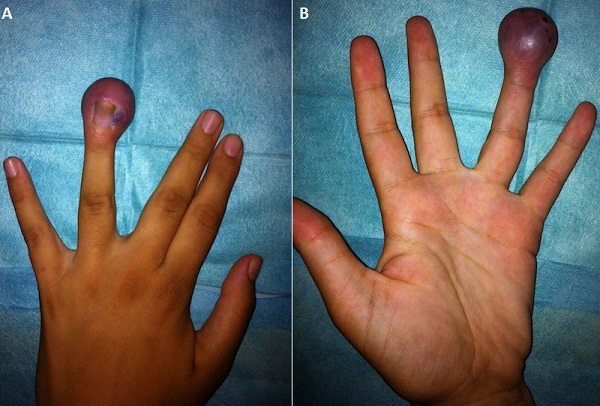
(A) face postérieure de la main gauche où il y a la malformation; (B) face antérieure de la main gauche avec la tuméfaction

